# Frequent physical activity is associated with reduced risk of severe diabetic retinopathy in type 1 diabetes

**DOI:** 10.1007/s00592-019-01454-y

**Published:** 2019-11-20

**Authors:** Heidi Tikkanen-Dolenc, Johan Wadén, Carol Forsblom, Valma Harjutsalo, Lena M. Thorn, Markku Saraheimo, Nina Elonen, Kustaa Hietala, Paula Summanen, Heikki O. Tikkanen, Per-Henrik Groop, the FinnDiane Study Group

**Affiliations:** 1 Folkhälsan Institute of Genetics, Folkhälsan Research Center, Biomedicum Helsinki, Helsinki, Finland; 2 Abdominal Center Nephrology, University of Helsinki and Helsinki University Hospital, Helsinki, Finland; 3 Research Programs Unit, Diabetes and Obesity, University of Helsinki, Helsinki, Finland; 4 The Chronic Disease Prevention Unit, National Institute for Health and Welfare, Helsinki, Finland; 5 Department of Ophthalmology, Helsinki University Hospital, Helsinki, Finland; 6 Department of Sports and Exercise Medicine, Institute of Clinical Medicine, University of Helsinki, Helsinki, Finland; 7 School of Medicine, Institute of Biomedicine, University of Eastern Finland, Kuopio, Finland; 8 Department of Diabetes, Central Clinical School, Monash University, Melbourne, VIC Australia

**Keywords:** Type 1 diabetes, Physical activity, Exercise, Retinopathy, Diabetic complications

## Abstract

**Aims:**

The aim of this study was to investigate whether leisure-time physical activity (LTPA) is associated with the development of severe diabetic retinopathy in individuals with type 1 diabetes.

**Methods:**

Prospective observational analysis as part of the Finnish diabetic nephropathy (FinnDiane) Study with a mean follow-up time of 10.7 years was performed. A total of 1612 individuals with type 1 diabetes were recruited, and LTPA was assessed at baseline using a validated self-report questionnaire. Severe diabetic retinopathy was defined as the initiation of laser treatment due to severe nonproliferative, proliferative retinopathy or diabetic maculopathy (identified from the Care Register for Health Care).

**Results:**

A total of 261 patients received laser treatment during the follow-up. Higher frequency of LTPA was associated with a lower incidence of severe diabetic retinopathy (*p* = 0.024), a finding that remained significant after adjustment for gender, duration, age at onset of diabetes, kidney function, BMI, triglycerides and systolic blood pressure. However, when HbA_1c_ and smoking were added to the Cox regression model the association was no more significant.

**Conclusions:**

Frequent LTPA is associated with a lower incidence of severe diabetic retinopathy during the follow-up. The total amount or the other components of LTPA (intensity or duration of a single session) were not associated with severe diabetic retinopathy.

**Electronic supplementary material:**

The online version of this article (10.1007/s00592-019-01454-y) contains supplementary material, which is available to authorized users.

## Introduction

Physical activity has been shown to be beneficial for individuals with type 1 diabetes and is associated with reduced risk of cardiovascular disease (CVD) events and premature mortality [1–6]. However, studies regarding physical activity and microvascular complications are limited in this patient group. This is particularly true for the relationship between physical activity and risk of diabetic retinopathy, and so far, no clear relationships have been found [5–7].

Among the studies that have addressed this issue, the Pittsburgh Insulin-Dependent Diabetes Mellitus Morbidity and Mortality Study made the first large attempt, but that study did not find any statistically significant associations between physical activity and diabetic retinopathy [5, 6]. However, the landmark Wisconsin Epidemiologic Study of Diabetic Retinopathy (WESDR) showed that participation in high school team sports was associated with a lower incidence of diabetic retinopathy, but only in a subgroup of women diagnosed with diabetes under the age of 14 [7].

In our previous study, we showed that a higher intensity of physical activity is associated with a lower risk of progression of diabetic nephropathy during the follow-up [8]. Notably, diabetic nephropathy and retinopathy represent common soil and share the same risk factors. In line with this, we previously showed that low-intensity LTPA was associated with proliferative retinopathy in a cross-sectional setting [9]. However, whether physical activity is beneficial also in individuals with type 1 diabetes with respect to diabetic retinopathy in a longitudinal setting is not clear. Therefore, the aim of this study was to assess whether baseline leisure-time physical activity (LTPA) and its components (intensity, duration and frequency) are associated with the development of severe diabetic retinopathy during the follow-up in individuals with type 1 diabetes.

## Methods

This prospective observational study is part of the ongoing Finnish Diabetic Nephropathy Study that has previously been described in detail [10]. The FinnDiane Study has recruited and thoroughly characterized more than 5000 individuals with type 1 diabetes at 92 centres throughout Finland. Included in the present study are those 1612 individuals with type 1 diabetes that had both LTPA and data on severe diabetic retinopathy available. Notably, patients that were blind (*n* = 1), had previously received laser treatment (*n* = 956) and had end-stage renal disease (*n* = 14) or unclear renal status at baseline were excluded (*n* = 79). At baseline, 41 patients had a major CVD event (myocardial infarction, coronary procedure or stroke). Type 1 diabetes was defined as a diagnosis of diabetes before the age of 40 years and permanent insulin treatment initiated within 1 year of the diagnosis. The study protocol was approved by the Ethical Committee of the Helsinki and Uusimaa Hospital District as well as by the local ethics committees at the participating centres and conducted according to the Declaration of Helsinki. All patients gave their written informed consent.

The primary study end point was severe diabetic retinopathy, defined as the initiation of laser treatment due to severe nonproliferative retinopathy, proliferative retinopathy or diabetic maculopathy identified from the Care Register for Health Care (HILMO).

Renal function (eGFR) was estimated using the CKD-EPI equation [11]. Renal status was categorized based on the urinary albumin excretion rate (AER) in either a timed overnight or 24-h urine collection in at least two out of three consecutive measurements as follows: normal AER, < 20 μg/min or < 30 mg/24 h (*n* = 1396); microalbuminuria, ≥ 20 and < 200 μg/min or ≥ 30 and < 300 mg/24 h (*n* = 154); or macroalbuminuria, ≥ 200 μg/min or ≥ 300 mg/24 h (*n* = 62). End-stage renal disease (ESRD; *n* = 14) was defined as ongoing dialysis or having received a previous kidney transplant. Blood pressure was measured after a 10-min rest twice in the sitting position with 2-min intervals, and the average of these two measurements was used in the analysis. Anthropometric data (weight, height, and waist and hip circumference) were recorded by a trained nurse. Fasting blood samples were drawn for the determination of HbA_1c_, lipid profile and serum creatinine. Smoking was assessed by a standardized questionnaire.

LTPA at baseline was assessed by a validated LTPA questionnaire. The questionnaire is based on the Kuopio Ischemic Heart Disease Risk Factor Study (KIHD) questionnaire that had been converted from the Minnesota LTPA questionnaire to a Finnish setting [13, 14]. The Minnesota LTPA questionnaire was validated by doubly labelled water and the KIHD LTPA questionnaire in turn by VO_2_ max [12–14]. The LTPA questionnaire contains information on the general type, frequency, intensity and duration of the patient’s physical activity recalled from the past 12 months. The total amount of LTPA is presented as METh/week, and patients were categorized as physically inactive (< 10 METh/week), moderately active (10–40 METh/week) and active (> 40 METh/week). In addition, LTPA intensity, single session duration and LTPA frequency were recorded. Patients were classified regarding these LTPA components as follows: intensity—low (no self-reported subjective shortness of breath and no sweating), moderate (a moderate degree of self-reported subjective shortness of breath and sweating) and high (a high degree of subjective shortness of breath and sweating); frequency—low (less than 1 session/week), moderate (1–2 sessions/week) and high (more than 2 sessions/week); duration—low (≤ 30 min/session), moderate (31–60 min/session) and high (> 60 min/session).

### Statistical analysis

The statistical analyses were conducted using the SPSS Statistics Software (version 22.2, IBM, Armonk, NY, USA). Comparisons between the groups were made using ANOVA for normally distributed variables and with the Kruskal–Wallis test for non-normally distributed variables. The chi-square test was used for categorical variables. Categorical variables are given as percentages. Normally distributed continuous variables are presented as mean ± SD, and those not normally distributed as median with interquartile range.

The cumulative incidence rates of severe diabetic retinopathy were assessed by the Kaplan–Meier method, and the logrank test was used to test the differences between groups. In the Kaplan–Meier analyses, the comparisons between the different levels of LTPA (low, moderate and high) were assessed by trend test when appropriate. The follow-up started from the baseline visit, and the person-years at risk were calculated until the first severe diabetic retinopathy event, death or the end of year 2015. The association between different LTPA components and severe diabetic retinopathy was analysed using Cox proportional univariable and multivariable hazard regressions. A *p* value less than 0.05 was considered statistically significant.

## Results

The total number of individuals studied was 1612, and the mean follow-up time was 10.7 ± 4.6 years. Of the whole population, 44.7% were men, mean age was 37.0 ± 11.9 years, mean duration of diabetes 18.9 ± 11.7 years, mean systolic blood pressure (SBP) 131 ± 16 mmHg, mean BMI 25.1 ± 3.6, mean HbA_1c_ 66.3 ± 14.9 mmol/mol (8.2 ± 1.4%), median LTPA 17.7 (8.3–33.5) METh/week, and 41.5% of the studied individuals were previous or current smokers.

The baseline clinical characteristics regarding total LTPA are shown in Table 1. Physically inactive patients were more often men and had a history of smoking. They had also worse glycaemic control and lipid profile and were more often obese. Table 2 presents the baseline clinical characteristics regarding the development of severe diabetic retinopathy. At baseline, the patients that developed severe retinopathy were younger and had higher blood pressure and BMI, worse lipid profile, higher HbA_1c_, more often a history of smoking and more frequent use of antihypertensive drugs.

**Table 1:** Baseline clinical characteristics stratified by LTPA

	Physically inactive	Moderately active	Active	*p* value
*N*	482	833	297	
Sex (men %)	50.2	41.1	46.1	0.005
Age (years)	36.9 ± 12.3	37.2 ± 11.7	36.7 ± 11.8	0.755
Duration of diabetes (years)	18.6 ± 11.8	19.1 ± 11.6	19.1 ± 11.7	0.722
BMI (kg/m^2^)	25.4 ± 3.8	25.1 ± 3.5	25.0 ± 3.1	0.145
WHR men	0.91 ± 0.07	0.90 ± 0.07	0.89 ± 0.06	0.013
WHR women	0.83 ± 0.06	0.81 ± 0.06	0.81 ± 0.07	0.021
SBP (mmHg)	132 ± 16	131 ± 16	131 ± 16	0.766
DBP (mmHg)	79 ± 10	79 ± 9	78 ± 9	0.850
HbA_1c_ (%)	8.4 ± 1.4	8.1 ± 1.3	8.2 ± 1.4	0.001
HbA_1c_ (mmol/mol)	68 ± 15	65 ± 15	66 ± 16	0.001
Total cholesterol (mmol/l)	4.80 ± 0.83	4.78 ± 0.86	4.75 ± 0.89	0.766
HDL cholesterol (mmol/l)	1.42 ± 0.40	1.49 ± 0.39	1.47 ± 0.42	0.014
LDL cholesterol (mmol/l)	2.86 ± 0.76	2.83 ± 0.78	2.78 ± 0.79	0.463
Triglycerides (mmol/l)	1.02 (0.76–1.46)	0.92 (0.71–1.26)	0.93 (0.71–1.32)	0.001
AHT (%)	25.3	20.8	22.6	0.173
Beta-blockers (%)	6.3	5.0	4.7	0.529
Ever-smokers (%)	51.4	39.7	37.8	< 0.001

Data are mean ± SD, median (IQR) or percentages

*p* values denote comparisons over three groups

*WHR* Waist-to-hip ratio; *AHT* antihypertensive medication; *SBP* systolic blood pressure; *DBP* diastolic blood pressure; *LTPA* leisure-time physical activity

**Table 2:** Baseline clinical characteristics regarding development of severe diabetic retinopathy

	No retinopathy	Retinopathy	*p* value
*N*	1351	261	
Sex (men %)	44.3	48.1	0.261
Age (years)	37.3 ± 11.9	35.6 ± 11.7	0.036
Duration of diabetes (years)	18.7 ± 12.0	19.8 ± 9.8	0.160
BMI (kg/m^2^)	25.1 ± 3.6	25.5 ± 3.5	0.048
WHR men	0.90 ± 0.07	0.90 ± 0.07	0.755
WHR women	0.82 ± 0.06	0.82 ± 0.06	0.211
SBP (mmHg)	131 ± 16	134 ± 17	0.003
DBP (mmHg)	78 ± 9	80 ± 10	0.001
HbA_1c_ (%)	8.0 ± 1.2	9.1 ± 1.6	< 0.001
HbA_1c_ (mmol/mol)	64 ± 14	76 ± 17	< 0.001
Total cholesterol (mmol/l)	4.75 ± 0.84	4.94 ± 0.89	0.001
HDL cholesterol (mmol/l)	1.49 ± 0.40	1.37 ± 0.40	< 0.001
LDL cholesterol (mmol/l)	2.80 ± 0.77	2.98 ± 0.79	0.001
Triglycerides (mmol/l)	0.92 (0.71–1.28)	1.04 (0.82–1.53)	< 0.001
AHT (%)	20.8	31.3	< 0.001
Beta-blockers (%)	5.3	5.4	0.944
Ever-smokers (%)	41.5	49.2	0.023
eGFR	99 ± 21	99 ± 21	0.941

Data are mean ± SD, median (IQR) or percentages

*WHR* Waist-to-hip ratio; *AHT* antihypertensive medication; *SBP* systolic blood pressure; *DBP* diastolic blood pressure; *LTPA* leisure-time physical activity

During the follow-up, a total of 261 of the patients developed severe diabetic retinopathy. The 10-year cumulative incidence rates for all LTPA components are shown in Table 3. In these Kaplan–Meier analyses, only frequency of physical activity was associated with a lower incidence of severe diabetic retinopathy during the follow-up.

**Table 3:** The 10-year cumulative incidence rates and 95% CI for the development of severe diabetic retinopathy by LTPA as well as by LTPA intensity, duration and frequency

	LTPA	Intensity	Frequency	Duration
Low*	15.6% (12.6,18.5) *N* = 482, 86 events	13.3% (9.9,16.7) *N* = 331,52 events	15.8% (12.3, 19.2) *N* = 328, 62 events	14.1% (9.4,18.5) *N* = 187, 30 events
Moderate*	13.7% (11.2,15.6) *N* = 833, 132 events	14.1% (11.9,16.3) *N* = 857,137 events	15.8% (12.6, 18.9) *N* = 420, 74 events	14.0% (11.8,16.2) *N* = 820, 128 events
High*	13.1% (9.4,17.1) *N* = 297, 43 events	15.0% (11.8,18.1) *N* = 388, 67 events	12.7% (10.6,14.7) *N* = 847,123 events	14.7% (11.8,17.5) *N* = 491, 84 events
*p* value	*P* = 0.12	*p* = 0.64	*p* = 0.024	*p* = 0.84

*LTPA* leisure-time physical activity

*LTPA: *Low *< 10 METh/week; *moderate* 10–40 METh/week and *high *> 40 METh/week; intensity: *low* (no self-reported subjective shortness of breath and no sweating); *moderate* (a moderate degree of self-reported subjective shortness of breath and sweating); and *high* (a high degree of subjective shortness of breath and sweating). Duration: *low* ≤ 30, *moderate* 31–60 and *high* > 60 min/session. Frequency: *low* < 1, *moderate* 1–2 and *high* > 2 sessions/week

The multivariable analyses regarding total LTPA, the LTPA components and the development of severe diabetic retinopathy are shown in Table 4. Model 1 is a univariable Cox regression model for the LTPA components and diabetic retinopathy. In the first model, the LTPA frequency was associated with a lower incidence of severe diabetic retinopathy during the follow-up. Next, we added the static risk factors to model 1: gender, duration, age at onset of diabetes and eGFR. After adjustment for these static confounders, LTPA frequency was still associated with severe diabetic retinopathy. Next, we added the dynamic risk factors triglycerides, systolic blood pressure, BMI and HbA_1c_ to model 3, and the association between LTPA frequency and severe diabetic retinopathy was no more significant. The final Cox regression model included the previous confounders and history of smoking. There was no association with severe diabetic retinopathy in this model. Notably, HbA_1c_ and history of smoking showed a strong association with the development of severe diabetic retinopathy. Interestingly, after adjustment for the static risk factors (model 2) as well as BMI, triglycerides and systolic blood pressure, the frequency of physical activity was still associated with severe diabetic retinopathy (*p* = 0.045). However, the association was no more significant after adding either history of smoking or HbA_1c_ to the model. The total amount of LTPA or the other components (intensity and duration) showed no association either in univariable or in multivariable models.

**Table 4:** Cox regression models showing hazard ratios (HRs) regarding severe diabetic retinopathy for low and moderate versus high total LTPA, intensity, frequency and duration

	LTPA	Intensity	Frequency	Duration
Model 1: low, moderate, high	1.31 (0.91–1.89) 1.10 (0.78–1.55) 1.00, *N* = 1612	0.92 (0.64–1.33) 0.92 (0.69–1.23) 1.00, *N* = 1576	1.40 (1.03–1.89) 1.24 (0.93–1.66) 1.00, *N* = 1595	0.94 (0.62–1.43) 0.92 (0.70–1.21) 1.00, *N* = 1498
Model 2: Model 1 + gender, duration of diabetes, age at onset of diabetes, kidney function	1.32 (0.91–1.90) 1.12 (0.80–1.59) 1.00, *N* = 1609	1.01 (0.69–1.47) 1.02 (0.75–1.38) 1.00, *N* = 1573	1.41 (1.04–1.92) 1.21 (0.90–1.62) 1.00, *N* = 1592	0.97 (0.64–1.48) 0.98 (0.74–1.30) 1.00, *N* = 1495
Model 3: Model 2 +SBP, TG, BMI, HbA_1c_	1.27 (0.88–1.85) 1.34 (0.94–1.91) 1.00, *N* = 1565	0.93 (0.63–1.38) 1.03 (0.76–1.41) 1.00, *N* = 1529	1.25 (0.91–1.71) 1.21 (0.90–1.63) 1.00, *N* = 1548	1.00 (0.65–1.54) 1.12 (0.83–1.47) 1.00, *N* = 1452
Model 4: Model 3 + history of smoking	1.22 (0.83–1.77) 1.32 (0.92–1.88) 1.00, *N* = 1518	0.82 (0.56–1.23) 0.98 (0.72–1.34) 1.00, *N* = 1482	1.18 (0.85–1.62) 1.21 (0.90–1.63) 1.00, *N* = 1502	0.96 (0.62–1.48) 1.08 (0.81–1.44) 1.00, *N* = 1408

*LTPA* leisure-time physical activity; *SBP* systolic blood pressure; *TG* triglycerides

We also performed the same analyses separately for individuals with normal AER. There was no association with LTPA or its components with severe diabetic retinopathy in this subgroup (*N* = 1396). However, the number of patients with severe diabetic retinopathy in this particular group was small (*N* = 190), and statistical power rather low. We also performed the same analyses in patients (*N* = 179) with normal AER, HbA1c < 8%, SBP < 135, BMI < 25 and with no history of smoking. In this subgroup, neither LTPA frequency nor the total amount of LTPA was associated with the development of severe diabetic retinopathy. This might be due to reduced power. Also, when the analyses were conducted in men and women separately, no associations were found.

## Discussion

In this prospective observational study, we show that frequency of LTPA is associated with a lower incidence of severe diabetic retinopathy in type 1 diabetes. The finding was unaffected by adjustment for gender, renal function, age at onset and duration of diabetes, BMI, systolic blood pressure or triglycerides. However, after adjustment for HbA_1c_ or history of smoking the association was no more significant. Neither total LTPA, nor single session duration nor intensity was associated with severe diabetic retinopathy during the follow-up.

To our knowledge, this is the first time an association between physical activity and severe diabetic retinopathy has been shown in an entire cohort of individuals with type 1 diabetes, even after adjustment for a number of confounders. So far, the only prospective studies that have assessed the relationship between physical activity and diabetic retinopathy are the Pittsburgh and the WESDR studies [5–7]. The Pittsburgh Insulin-Dependent Diabetes Mellitus Morbidity and Mortality Study reported a trend towards a lower risk of severe diabetic retinopathy in individuals who participated in team sports. The findings were, however, not statistically significant. A further 5-year follow-up of the same cohort revealed similar findings. In men, physical activity during high school was associated with less signs of retinopathy, but adjustment for duration of diabetes eliminated the relationship. [5, 6] Importantly, these earlier studies showed no negative effect of physical activity on the development of severe diabetic retinopathy. The WESDR showed in women with diabetes diagnosis before the age of 14 years that participation in team sports during high school or college was associated with a lower incidence of proliferative diabetic retinopathy. This relationship remained significant when adjusted for age and duration of diabetes. However, in men, or in other patients in the WESDR cohort no such associations were found. Our study extends these previous results to both genders and suggests that there is an association between physical activity and lower incidence of severe diabetic retinopathy.

In our previous study on physical activity and diabetic nephropathy, the intensity seemed to be the most important component of LTPA for the progression of kidney disease [8]. Since diabetic nephropathy is closely linked to diabetic retinopathy, we assumed that the intensity would also be associated with the risk of severe diabetic retinopathy. Such a view was supported by our previous cross-sectional data, showing that the intensity was indeed associated with diabetic retinopathy [9]. To our surprise, only the frequency of LTPA showed an association in this longitudinal data set. In fact, in the higher-intensity groups, the cumulative incidence of severe diabetic retinopathy was even higher, although not significant. The reason for this is unknown, but it can be assumed that a more precise retinopathy status is required to be able to assess the relationship, potential drawbacks or benefits between intensive physical activity and diabetic retinopathy. The current ADA recommendations for diabetes and physical activity state that: “Vigorous aerobic or resistance exercise; jumping, jarring, head-down activities; and breath holding should be avoided in anyone with severe non-proliferative and unstable proliferative diabetic retinopathy” [15]. Notably, our observation suggests that these recommendations are well grounded. It has been shown that autoregulation is disturbed in patients with diabetes and especially if the patient has poor glycaemic control and microvascular impairment [16]. In such patients, intensive exercise and the increase in blood pressure may lead to retinal damage due to even further impaired autoregulation. On the other hand, further studies are needed to assess whether physical activity and potential hemodynamic adaptations could lead to improved vascular reactivity [17]. The finding regarding the potential benefit of frequent physical activity is also in line with the current ADA recommendations for physical activity in individuals with type 1 diabetes [15]. We cannot totally exclude any relationship between the other components of LTPA given the rather crude dichotomous classification of severe diabetic retinopathy (yes/no), which may reduce our possibility to demonstrate possible associations. The use of the EDTRS scale, as well as thorough characterization of the retinal status by optical coherence tomography (OCT), could possibly help to identify those individuals that might benefit and those that might be at risk of further damage by physical activity.

In type 1 diabetes, glycaemic control, blood pressure and triglycerides are treatable or dynamic risk factors for diabetic retinopathy as shown in randomized controlled trials [18–22]. There is some evidence that smoking and obesity might also be treatable risk factors for diabetic retinopathy [23–26]. Therefore, it is of note that there are several potential mechanisms, related to these factors, how physical activity might be beneficial for the prevention of diabetic retinopathy. In our multivariable analysis, the adjustment for HbA_1c_ abolished the association with frequent physical activity. This might be due to a beneficial effect of physical activity on HbA_1c_. Furthermore, physical activity has been shown to improve insulin sensitivity in type 1 diabetes, but the evidence regarding HbA_1c_ lowering is so far controversial [27]. This might be because HbA_1c_ may not be an ideal indicator of glycaemic control, since it does not reflect the glycaemic variability. Physical activity in general improves the lipid profile, BMI and blood pressure. However, in type 1 diabetes only the improvement in lipid levels has been established [28]. Since these dynamic factors did not mitigate the association of frequent physical activity with severe diabetic retinopathy in the multivariable analysis, the data suggest that a possible beneficial effect might be mediated through other factors. For example, it is well known that physical activity improves vascular endothelial function and reduces inflammation [28, 29].

An important strength of our study is the prospective study design including a large nationwide cohort of individuals with type 1 diabetes. In addition, due to the nationwide registries there were no patients lost to follow-up. Given the size and the comprehensive nationwide approach, our study adds important information to the limited availability of data on physical activity and diabetic retinopathy in type 1 diabetes. Importantly, we used a previously validated LTPA questionnaire capturing data not only on total LTPA but also on the various LTPA components, intensity, duration and frequency.

However, there are also some limitations. Physical activity was assessed by a self-report questionnaire, and work-related physical activity was not evaluated, a fact that could lead to a potential over- or underestimation of the true physical activity level. In addition, we could not assess the specific type of exercise (such as isotonic vs. isometric) that theoretically could affect the pathogenesis of diabetic retinopathy. More precise objective physical activity assessment tools are not feasible for such large nationwide cohorts as ours, and they have their own potential bias such as increased activity during surveillance. Some potential confounders such as nutrition and socio-economic status were not either evaluated in our study. Instead, we used smoking as a confounder as smoking has been shown to be strongly correlated with the socio-economic status [30, 31]. The development of severe diabetic retinopathy was assessed by information on laser treatment obtained from care registers rather than from a review of fundus photographs. Fundus photography would of course have given a more precise assessment of the disease status, and this limitation might have resulted in potential disease misclassification both at baseline and at follow-up. However, if anything, this crude classification may only have diluted the findings.

In conclusion, frequent LTPA was associated with a lower incidence of severe diabetic retinopathy in type 1 diabetes during the follow-up. The total amount of LTPA or its components (intensity or the duration of a single session) were not associated with severe diabetic retinopathy.

## Electronic supplementary material

Below is the link to the electronic supplementary material.

Supplementary material 1 (DOCX 32 kb)
